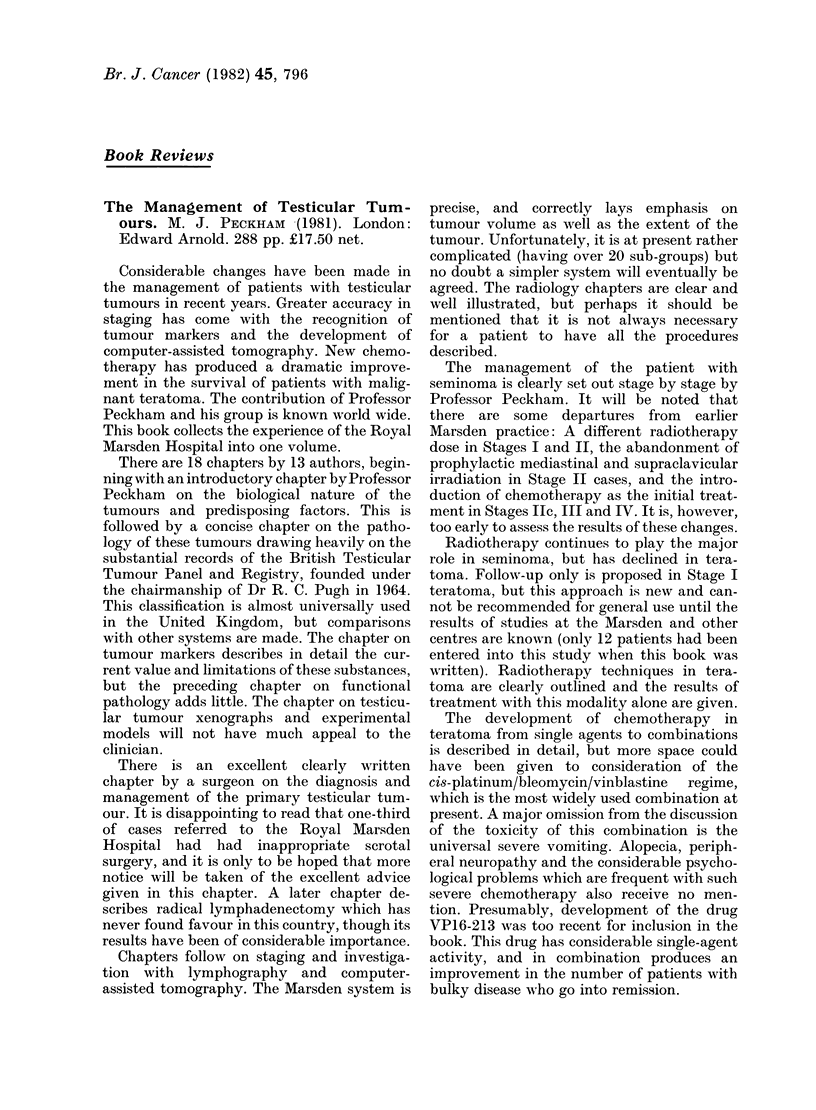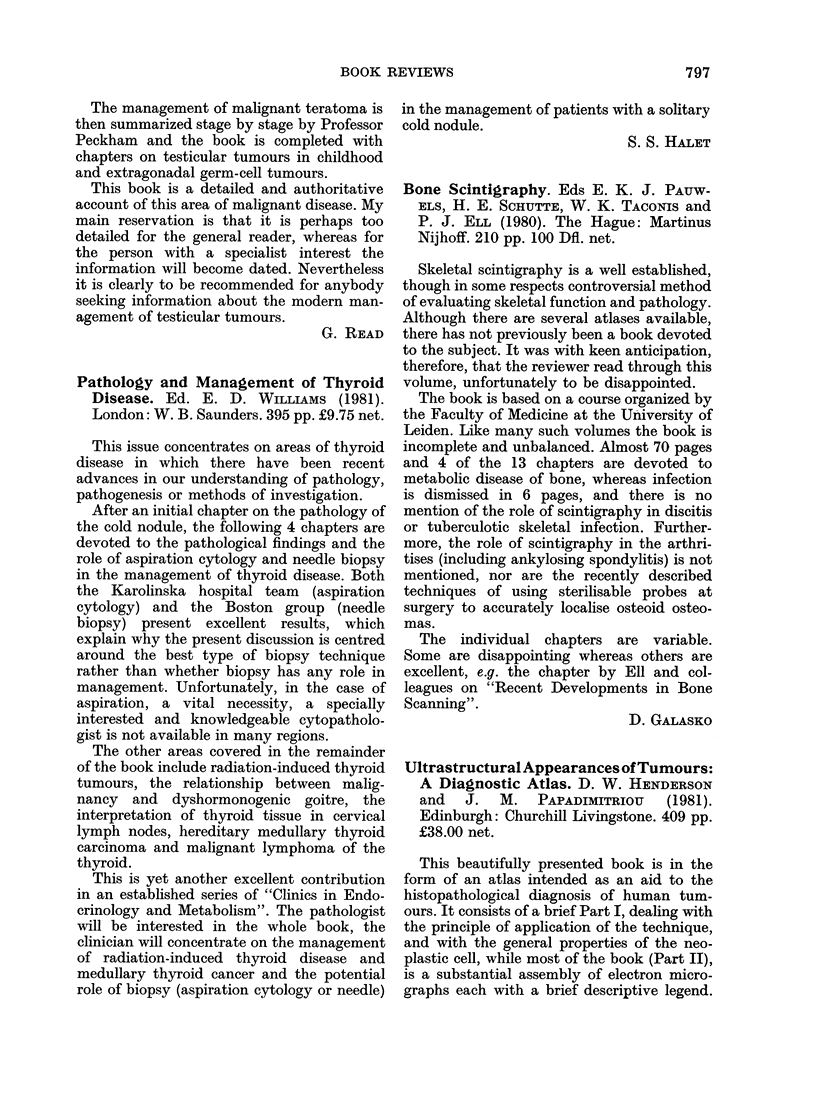# The Management of Testicular Tumours

**Published:** 1982-05

**Authors:** G. Read


*Br. J. Cancer* (1982) **45**, 796

### *Book Reviews*

**The Management of Testicular Tumours.** M. J. PECKHAM (1981). London: Edward Arnold. 288 pp. £17.50 net.

Considerable changes have been made in the management of patients with testicular tumours in recent years. Greater accuracy in staging has come with the recognition of tumour markers and the development of computer-assisted tomography. New chemotherapy has produced a dramatic improvement in the survival of patients with malignant teratoma. The contribution of Professor Peckham and his group is known world wide. This book collects the experience of the Royal Marsden Hospital into one volume.

There are 18 chapters by 13 authors, beginning with an introductory chapter by Professor Peckham on the biological nature of the tumours and predisposing factors. This is followed by a concise chapter on the pathology of these tumours drawing heavily on the substantial records of the British Testicular Tumour Panel and Registry, founded under the chairmanship of Dr R. C. Pugh in 1964. This classification is almost universally used in the United Kingdom, but comparisons with other systems are made. The chapter on tumour markers describes in detail the current value and limitations of these substances, but the preceding chapter on functional pathology adds little. The chapter on testicular tumour xenographs and experimental models will not have much appeal to the clinician.

There is an excellent clearly written chapter by a surgeon on the diagnosis and management of the primary testicular tumour. It is disappointing to read that one-third of cases referred to the Royal Marsden Hospital had had inappropriate scrotal surgery, and it is only to be hoped that more notice will be taken of the excellent advice given in this chapter. A later chapter describes radical lymphadenectomy which has never found favour in this country, though its results have been of considerable importance.

Chapters follow on staging and investigation with lymphography and computer-assisted tomography. The Marsden system is precise, and correctly lays emphasis on tumour volume as well as the extent of the tumour. Unfortunately, it is at present rather complicated (having over 20 sub-groups) but no doubt a simpler system will eventually be agreed. The radiology chapters are clear and well illustrated, but perhaps it should be mentioned that it is not always necessary for a patient to have all the procedures described.

The management of the patient with seminoma is clearly set out stage by stage by Professor Peckham. It will be noted that there are some departures from earlier Marsden practice: A different radiotherapy dose in Stages I and II, the abandonment of prophylactic mediastinal and supraclavicular irradiation in Stage II cases, and the introduction of chemotherapy as the initial treatment in Stages IIc, III and IV. It is, however, too early to assess the results of these changes.

Radiotherapy continues to play the major role in seminoma, but has declined in teratoma. Follow-up only is proposed in Stage I teratoma, but this approach is new and cannot be recommended for general use until the results of studies at the Marsden and other centres are known (only 12 patients had been entered into this study when this book was written). Radiotherapy techniques in teratoma are clearly outlined and the results of treatment with this modality alone are given.

The development of chemotherapy in teratoma from single agents to combinations is described in detail, but more space could have been given to consideration of the *cis*-platinum/bleomycin/vinblastine regime, which is the most widely used combination at present. A major omission from the discussion of the toxicity of this combination is the universal severe vomiting. Alopecia, peripheral neuropathy and the considerable psychological problems which are frequent with such severe chemotherapy also receive no mention. Presumably, development of the drug VP16-213 was too recent for inclusion in the book. This drug has considerable single-agent activity, and in combination produces an improvement in the number of patients with bulky disease who go into remission.

The management of malignant teratoma is then summarized stage by stage by Professor Peckham and the book is completed with chapters on testicular tumours in childhood and extragonadal germ-cell tumours.

This book is a detailed and authoritative account of this area of malignant disease. My main reservation is that it is perhaps too detailed for the general reader, whereas for the person with a specialist interest the information will become dated. Nevertheless it is clearly to be recommended for anybody seeking information about the modern management of testicular tumours.

G. READ